# miR-296-3p, miR-298-5p and their downstream networks are causally involved in the higher resistance of mammalian pancreatic α cells to cytokine-induced apoptosis as compared to β cells

**DOI:** 10.1186/1471-2164-14-62

**Published:** 2013-01-29

**Authors:** Davide Barbagallo, Salvatore Piro, Angelo G Condorelli, Loriana G Mascali, Francesca Urbano, Nunziatina Parrinello, Adelina Monello, Luisa Statello, Marco Ragusa, Agata M Rabuazzo, Cinzia Di Pietro, Francesco Purrello, Michele Purrello

**Affiliations:** 1 Dipartimento Gian Filippo Ingrassia, Unità di BioMedicina Molecolare Genomica e dei Sistemi Complessi, Genetica, Biologia Computazionale, Università di Catania, Catania, EU 95123, Italy; 2 Dipartimento di BioMedicina Clinica e Molecolare, Università di Catania, Catania, EU 95122, Italy

**Keywords:** Mammalian pancreatic α and β cells, microRNA transcriptome, Proinflammatory cytokines, Apoptosis, Cellular networks, Diabetes mellitus

## Abstract

**Background:**

The molecular bases of mammalian pancreatic α cells higher resistance than β to proinflammatory cytokines are very poorly defined. MicroRNAs are master regulators of cell networks, but only scanty data are available on their transcriptome in these cells and its alterations in diabetes mellitus.

**Results:**

Through high-throughput real-time PCR, we analyzed the steady state microRNA transcriptome of murine pancreatic α (αTC1-6) and β (βTC1) cells: their comparison demonstrated significant differences. We also characterized the alterations of αTC1-6 cells microRNA transcriptome after treatment with proinflammatory cytokines. We focused our study on two microRNAs, miR-296-3p and miR-298-5p, which were: (1) specifically expressed at steady state in αTC1-6, but not in βTC1 or INS-1 cells; (2) significantly downregulated in αTC1-6 cells after treatment with cytokines in comparison to untreated controls. These microRNAs share more targets than expected by chance and were co-expressed in αTC1-6 during a 6–48 h time course treatment with cytokines. The genes encoding them are physically clustered in the murine and human genome. By exploiting specific microRNA mimics, we demonstrated that experimental upregulation of miR-296-3p and miR-298-5p raised the propensity to apoptosis of transfected and cytokine-treated αTC1-6 cells with respect to αTC1-6 cells, treated with cytokines after transfection with scramble molecules. Both microRNAs control the expression of IGF1Rβ, its downstream targets phospho-IRS-1 and phospho-ERK, and TNFα. Our computational analysis suggests that MAFB (a transcription factor exclusively expressed in pancreatic α cells within adult rodent islets of Langerhans) controls the expression of miR-296-3p and miR-298-5p.

**Conclusions:**

Altogether, high-throughput microRNA profiling, functional analysis with synthetic mimics and molecular characterization of modulated pathways strongly suggest that specific downregulation of miR-296-3p and miR-298-5p, coupled to upregulation of their targets as IGF1Rβ and TNFα, is a major determinant of mammalian pancreatic α cells resistance to apoptosis induction by cytokines.

## Background

Insulitis is an inflammation of pancreatic Langerhans islets, which is known to precede the onset of diabetes mellitus (DM) [1,2]. Post-insulitis decrease of pancreatic β cells is a major hallmark of both type 1 (T1DM) and type 2 diabetes mellitus (T2DM) [3-5]. It is associated to α cells dysfunctions and high glucagon secretion, which contribute to chronic hyperglycemia and ensuing clinical outcomes in DM [6,7]. Accordingly, efforts to increase our knowledge on the biomolecular mechanisms regulating proliferation, physiopathological functions, and apoptosis of α cells will likely result in improved medical approaches to the disease. The pathways leading to mammalian β cell apoptosis, induced by proinflammatory cytokines, are complex but sufficiently known [8,9]. On the contrary, the molecular bases of α cells higher resistance than β to the same cues are still largely uncharacterized [10-12]. Pancreatic α and β cells share common endocrine precursors (Ngn3^+^ cells), but upon differentiation they respond differently to external stimuli as proinflammatory cytokines, glucagon-like peptide 1 (GLP1), and inhibitors of dipeptidyl peptidase-4 (DPP-4) [12-14]. MicroRNAs (miRNAs) are small (19–25 nucleotides) noncoding RNAs, which control gene expression mainly at the post-transcriptional level: miRNAs have been shown to be master regulators of cell networks [15,16]. Many miRNAs are involved in DM physiopathology: for instance, miR-375 controls β cell mass and insulin secretion [17]; miR-21 and miR-146a are involved in β-cell apoptosis [18]; miR-30d promotes insulin synthesis and protects β cells from damage by proinflammatory cytokines [19]; miR-126 is responsible for impaired angiogenetic signaling in DM patients [20]. However, there are no high-throughput (HT) published data on α cells miRNA transcriptome at steady state, its comparison with β cells steady state miRNA transcriptome, and its alterations following treatment with cytokines. To discover new physiopathologic regulatory mechanisms, specific of cytokine-treated pancreatic cells, we profiled global miRNAs expression in two mouse pancreatic cell lines (αTC1-6 and βTC1) at steady state and in αTC1-6 after treatment with proinflammatory cytokines IFN-γ, IL-1β, TNF-α. This analysis brought to the identification of: (1) differentially expressed (DE) miRNAs between αTC1-6 and βTC1 at steady state; (2) DE miRNAs between αTC1-6 treated with cytokines and matched untreated controls. To insert our data in the appropriate biological context, we reconstructed the molecular networks regulated at steady state by DE miRNAs in α and β cells. Our results demonstrate that these networks are remarkably different: very likely, they increase α cells resistance to apoptosis and negatively interfere with pancreatic β cells viability after exposure to cytokines.

## Results

### Apoptosis induction by cytokines in αTC1-6 and βTC1 cells

**Figure 1:**
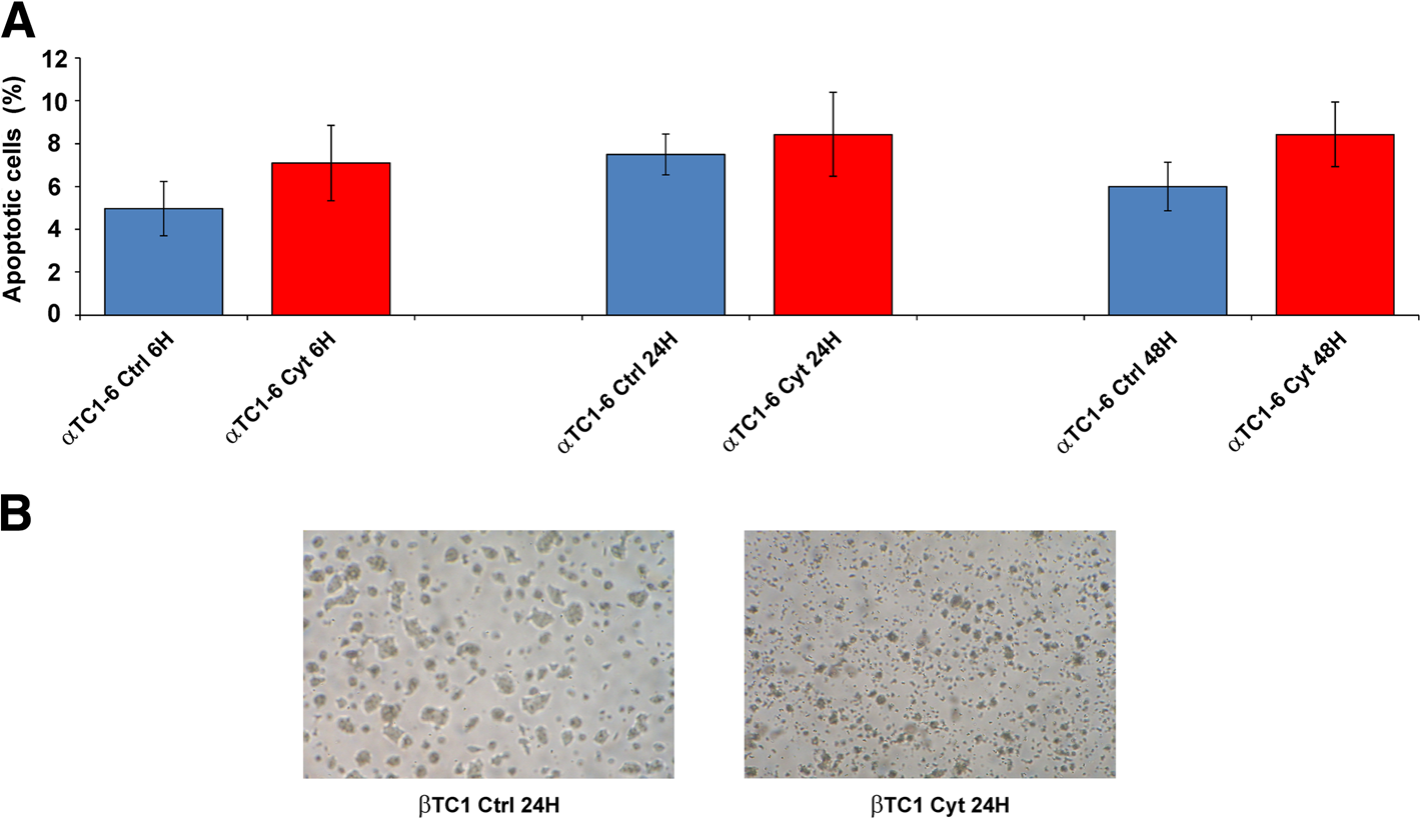
**Apoptosis of αTC1-6 and βTC1 after treatment with cytokines. (A)** Annexin V flow cytometric analysis of apoptosis in αTC1-6 treated with IFN-γ, IL-1β, TNF-α for 6, 24, 48 h and in matched untreated controls. Values represent the percentage of apoptotic cells. Data are presented as mean ± S.D. of three independent experiments (n = 3). **(B)** Microphotographs of βTC1 at steady state (left) and 24 h PT (right). Cell shrinkage and irregular morphology are evident in βTC1 after 24 h of treatment. Representative pictures are shown from three independent experiments (n = 3).

To verify the differential response of αTC1-6 and βTC1 cells to treatment with cytokines, we assayed their propensity to undergo apoptosis during a time course experiment (6-24-48 h after treatment, PT). The number of apoptotic αTC1-6 cells did not significantly vary with respect to controls for the entire time course (Figure 1A), demonstrating that in our system α cells appear to withstand apoptosis induction by cytokines; on the contrary, βTC1 cells are clearly more susceptible to treatment (Figure 1B).

### Steady state miRNA transcriptome profiles of αTC1-6 and βTC1 cells

To identify miRNAs potentially responsible for αTC1-6 resistance to cytokines, we analyzed the steady state miRNA transcriptome of αTC1-6 and βTC1 cells. At steady state, 23 miRNAs were exclusively expressed in αTC1-6 (α-miRNAs), while 26 were expressed only in βTC1 (β-miRNAs); 50 miRNAs resulted significantly more expressed in αTC1-6 than in βTC1, whereas 74 were significantly more expressed in βTC1 compared to αTC1-6 (Limma test, Benjamini-Hochberg adjusted p-values < 0.05) (see Additional file 1). Their assignment to specific families is shown in Additional files 2 and 3.

### Treatment with cytokines alters αTC1-6 miRNA transcriptome profiles

After treatment with cytokines for 48 h, 3 miRNAs (miR-146a, miR-203, miR-298-5p) were significantly differentially expressed in αTC1-6 as compared to matched untreated controls (Limma test, Benjamini-Hochberg adjusted p-values < 0.05) (see Additional file 4). Their assignment to specific families is shown in Additional file 5.

### MiRNAs 296-3p and 298-5p are reliable candidates for involvement in αTC1-6 higher resistance than β cells to apoptosis induction by cytokines

**Figure 2:**
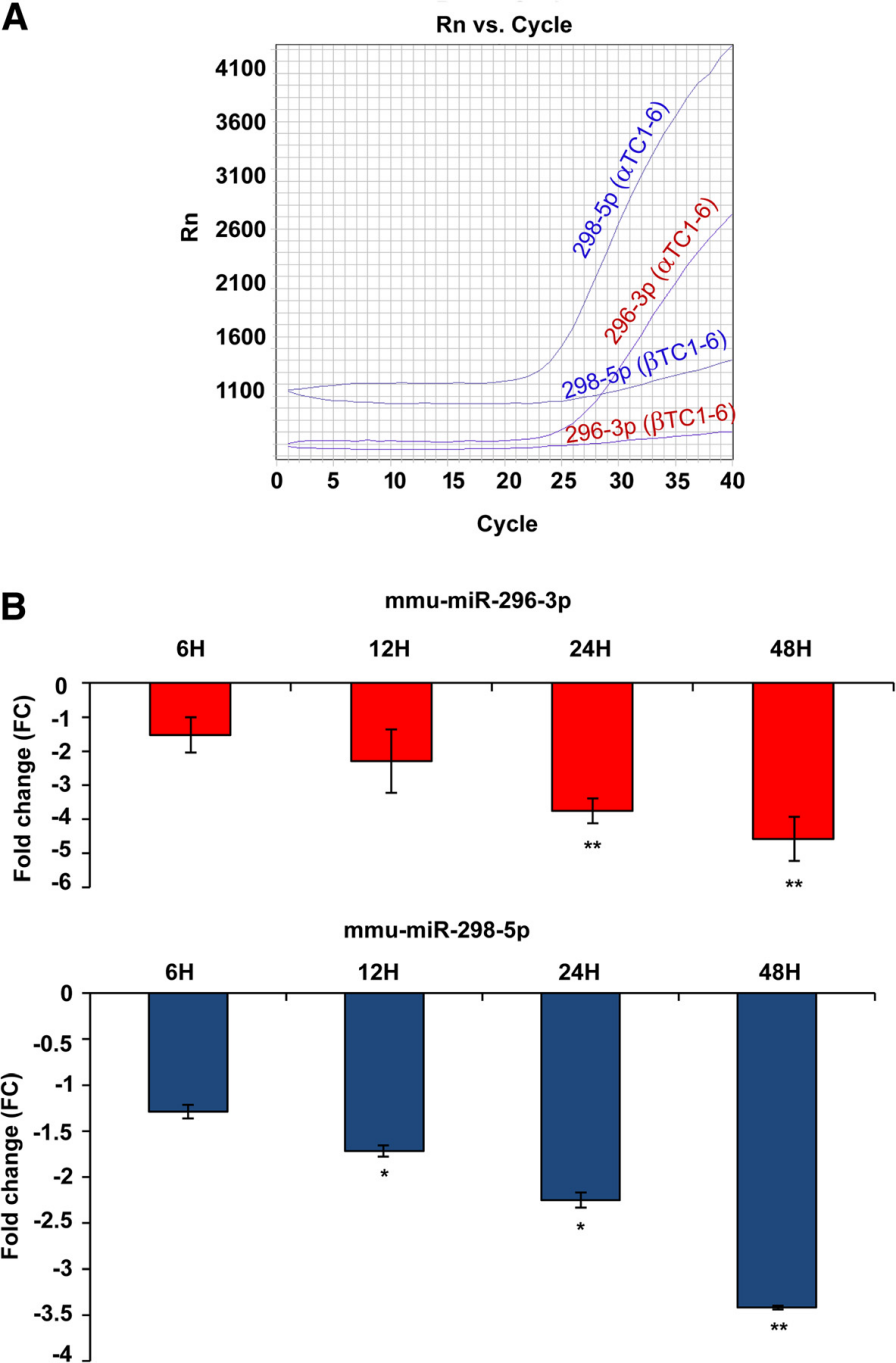
**Expression of miR-296-3p and miR-298-5p in αTC1-6 and βTC1. (A)** Real-time PCR amplification plot of miR-296-3p and miR-298-5p in αTC1-6 and βTC1 at steady state. Note that both miRNAs are not expressed in βTC1. Plot image is representative of three independent experiments (n = 3). **(B)** Gene expression fold changes of miR-296-3p and miR-298-5p in αTC1-6 at 6, 12, 24, 48 h PT, relative to matched untreated cells. MiRNAs expression was measured by quantitative real-time PCR. MiR-26a was used as endogenous control. Data are reported as mean ± S.D. of three independent experiments (n = 3). * p-value < 0.05; ** p-value < 0.01 (Student’s *t*-test, Bonferroni correction).

To identify miRNAs whose functions could explain the differential response to cytokines of pancreatic α and β cells, we specifically focused our attention on miR-296-3p and miR-298-5p. Both miRNAs are expressed at steady state only in αTC1-6, but are not synthesized in βTC1 (Figure 2A); both are part of the imprinted Gnas/GNAS clusters in mice and humans and share more targets than expected by chance (44 versus 33, respectively; p = 0.0485, *χ*^2^-test), even though their seed regions are different: this suggests common regulatory functions. Single TaqMan gene expression assays (STAs) during a time course analysis (6-12-24-48 h) showed a highly significant downregulation of miR-296-3p in αTC1-6 cells at 24 and 48 h PT, compared to matched untreated controls (Student’s *t*-test, Bonferroni adjusted p-value < 0.01). Mir-298-5p resulted significantly downregulated starting at 12 h PT and reached a highly significant downregulation at 48 h PT, compared to matched untreated controls: Student’s *t*-test, Bonferroni adjusted p-values, were <0.05 and <0.01, respectively (Figure 2B). Both miRNAs were co-expressed in αTC1-6 cells throughout the entire experimental time course (r-value = 0.88, p-value = 1.15e-08, Pearson’s correlation test) (see Additional file 6). STAs confirmed that they are not expressed either in βTC1 or INS-1 cells.

### Genomics of genes encoding miR-296-3p, miR-298-5p, *Nespas* and identification of upstream CpG islands

Genes encoding miRNAs 296-3p and 298-5p are clustered in a genomic region, which also comprises the gene for the noncoding transcript *Nespas* and is imprinted in mice and humans [21]. Sequences of mature miR-296-3p are 100% conserved between rodents and humans, whereas those of miR-298-5p are 74% identical. Analysis of this region through UCSC browser revealed the presence of two clusters of CpG islands: (i) one comprises two CpG islands, from 17.5 to 18.8 kb upstream the first nucleotide of pre-miR-296, and is located 9.2 and 10 Kb downstream *Nespas* transcription start site (TSS); (ii) the other is made of three CpG islands from 30.1 to 33.6 Kb upstream the first nucleotide of pre-miR-296 and is located 1.8-5 kb upstream *Nespas* TSS (see Additional file 7). MatInspector revealed a putative *Nespas* promoter located 500 bp upstream-100 bp downstream its TSS.

### αTC1-6 transfection with mimics of miR-296-3p and miR-298-5p increases apoptosis levels induced by cytokines

**Figure 3:**
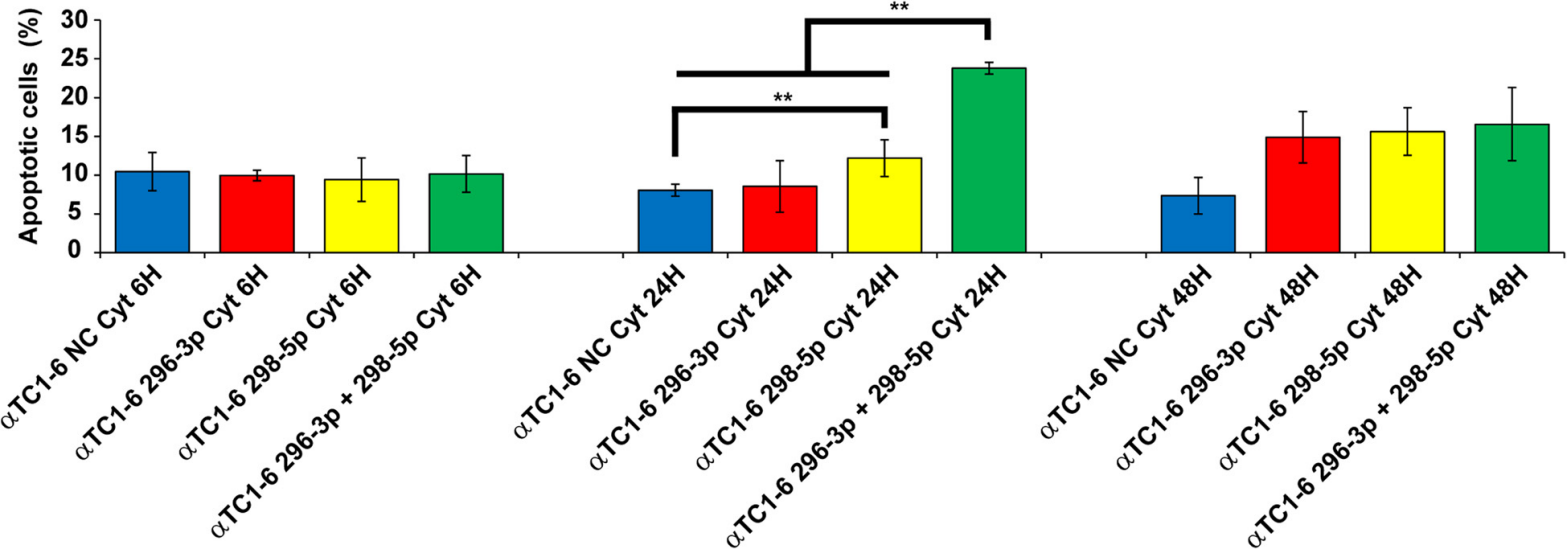
**Upregulation of miR-296-3p and miR-298-5p reduces αTC1-6 resistance to apoptosis induced by cytokines.** Annexin V flow cytometric analysis of apoptosis in αTC1-6 transiently transfected with scrambled oligonucleotides (NC, Negative Control), mimics of miR-296-3p, miR-298-5p, a mix of both, and treated with IFN-γ, IL-1β, TNF-α for 6, 24, 48 h. The y-axis represents the percentage of apoptotic cells; the x-axis represents the four experimental conditions [(i)scramble-transfected cells; (ii) cells transfected with mimics of miR-296-3p; (iii) cells transfected with mimics of miR-298-5p; (iv) cells transfected with a mix of both] assayed at the three time-points. Data are presented as mean ± S.D. of three independent experiments (n = 3). All the possible pairwise comparisons were performed among the four different experimental conditions within each time point: significant differences have been assessed through Tukey HSD post-hoc one-way ANOVA test (** p-value < 0.01). At 24 h PT, αTC1-6 transfected with mimics of miR-298-5p show a highly significant increase of the number of apoptotic cells with respect to scramble-transfected control; at the same time point, in αTC1-6 transfected with mimics of both miR-296-3p and miR-298-5p a highly significant increase of the number of apoptotic cells is detected with respect to all the other experimental conditions.

To precisely define miR-296-3p and miR-298-5p biological functions, we compared apoptosis levels of αTC1-6 cells, transfected with either one or both miRNA mimics and treated with cytokines for 6, 24, 48 h after transfection (AT), with those of scramble-transfected αTC1-6 cells treated with cytokines by following a similar protocol. The percentage of apoptotic αTC1-6 cells after transfection with mimics of miR-296-3p was comparable to scramble-transfected controls during the entire time course treatment. On the other hand, transfection with mimics of miR-298-5p or of both miR-296-3p and miR-298-5p increased in a highly significant manner the number of αTC1-6 apoptotic cells, compared to matched scramble-transfected controls: 1.5 and more than 2.5 folds at 24 h PT, respectively (Tukey HSD post-hoc one-way ANOVA test, p-value <0.01) (Figure 3).

### Identification of miR-296-3p and miR-298-5p targets

To characterize the networks regulated by miRNAs 296-3p and 298-5p, we computationally searched their validated and predicted targets. We identified 1 validated target of miR-296-3p; 5 validated targets of miR-298-5p; 207 predicted targets of miR-296-3p; 707 predicted targets of miR-298-5p. We focused our attention on 7 targets of miR-296-3p, 4 of miR-298-5p, 2 common to both miRNAs: they were chosen according to their involvement in apoptosis, cell cycle progression, cell differentiation and hormone secretion (see Additional file 8). Among them, *Bcl2*, *Ccna2*, *Irs2*, *Nr4a2* are transcriptionally regulated by CREB1, which is a validated target of miR-296-3p [22]; *Tnf* and *Vdr* are validated targets of miR-298-5p [23,24]. All other targets were computationally predicted, including IGF1Rβ that we validated through western analysis (see later).

### Modulation of miR-296-3p and miR-298-5p also alters expression of their targets

**Figure 4:**
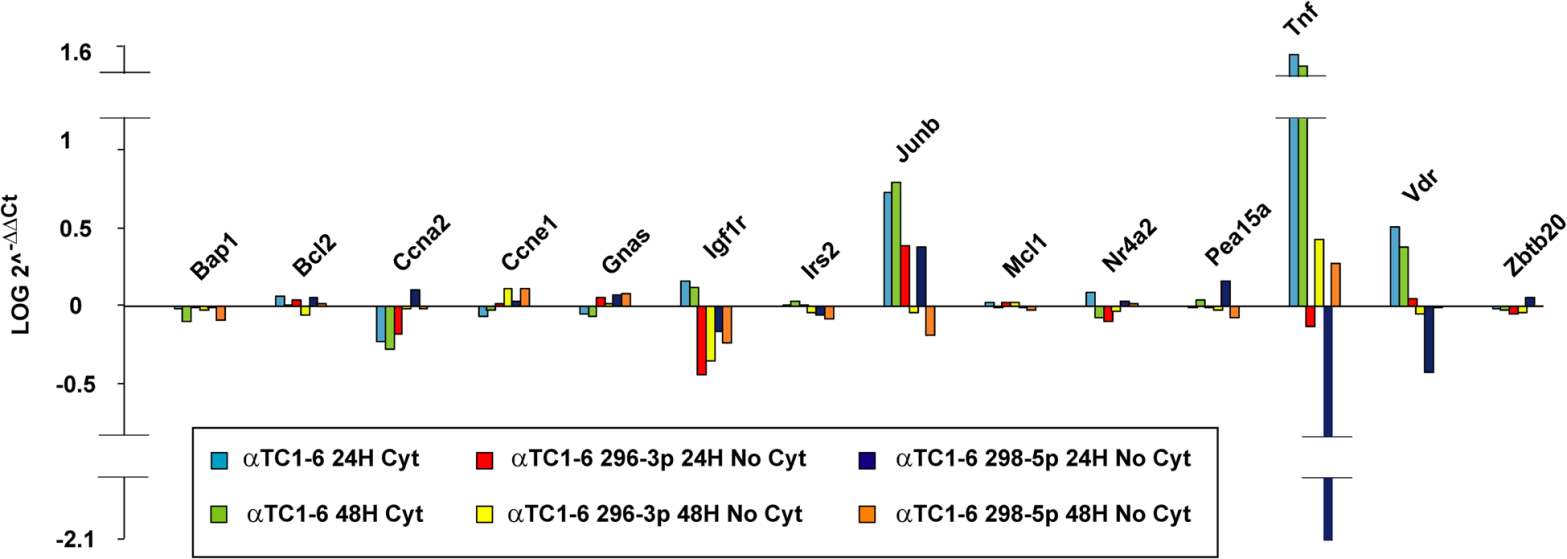
**Modulation of miR-296-3p and miR-298-5p alters expression of their targets.** Bar graph showing changes in gene expression of a selected set of miR-296-3p and miR-298-5p targets for each of three different experimental conditions: (i) αTC1-6 treated with cytokines with respect to matched untreated control cells at the time points 24 and 48 h; (ii) untreated αTC1-6 transfected with mimics of miR-296-3p with respect to scramble-transfected control cells at the time points 24 and 48 h; (iii) untreated αTC1-6 transfected with mimics of miR-298-5p with respect to scramble-transfected control cells at the time points 24 and 48 h. Data are reported as LOG of 2^^-ΔΔCt^ values. Hprt and Ppia were used as endogenous controls to normalize real-time PCR data.

To verify whether *in vitro* modulation of miR-296-3p and miR-298-5p affected the expression of their targets, we performed transient transfection experiments of αTC1-6 cells with their mimics. Transfection efficiency at 24 h AT was in all cases higher than 90%. In αTC1 transfected with mimics of miR-296-3p or miR-298-5p, real-time PCR showed altered expression of different genes with respect to scramble-transfected cells, including *Igf1r*, *Tnf*, *Vdr* (Figure 4).

### Expression of IGF1Rβ and TNFα is controlled by miR-296-3p and miR-298-5p in αTC1-6 cells

**Figure 5:**
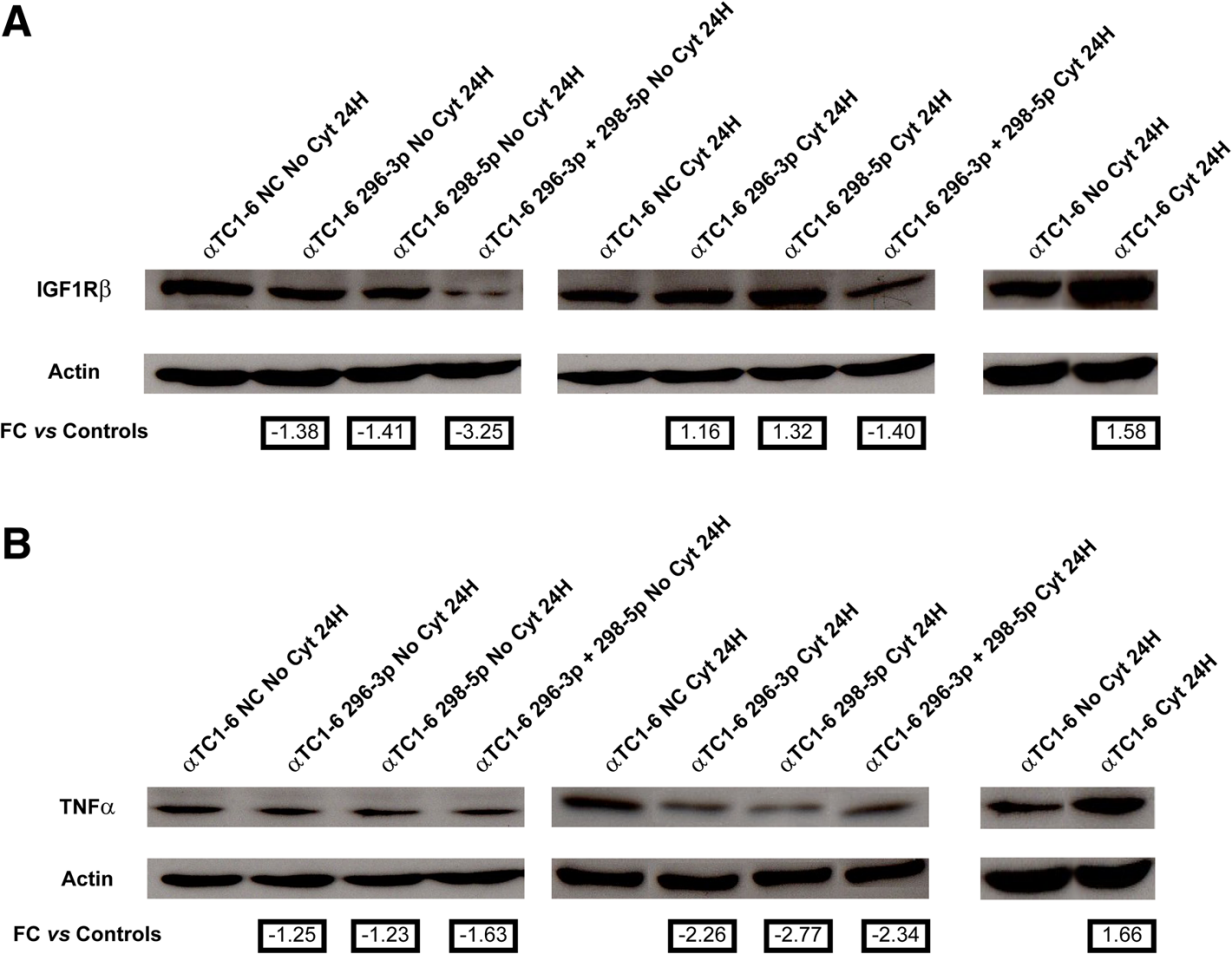
**Expression of IGF1Rβ and TNFα proteins is regulated by miR-296-3p and miR-298-5p in αTC1-6.** (**A**) Western analysis of IGF1Rβ in (1) untreated αTC1-6 transfected for 24 h with (i) scramble molecules (NC); (ii) mimics of miR-296-3p; (iii) mimics of miR-298-5p; (iv) mimics of both miR-296-3p and miR-298-5p (left); (2) αTC1-6 transfected for 24 h with (i) scramble molecules (NC); (ii) mimics of miR-296-3p; (iii) mimics of miR-298-5p; (iv) mimics of both miR-296-3p and miR-298-5p and treated with cytokines for further 24 h (middle); (3) αTC1-6 treated with cytokines for 24 h and their matched untreated controls (right). **(B)** Western analysis of TNF-α performed in the same experimental conditions as (**A**). β-Actin signal was used to normalize the data. Numbers below Actin blots represent fold change expression values relative to matched controls.

In αTC1-6 treated with cytokines for 24 h, levels of proteins IGF1Rβ and TNFα increased 1.5 and 1.7 folds with respect to untreated controls, respectively (Figures 5A and 5B, right panels), while miRNAs 296-3p and 298-5p decreased of 3.7 and 2.2 folds with respect to untreated controls, respectively (Figure 2B). We further demonstrated through western analysis that protein IGF1Rβ decreased about 1.4 folds in steady-state αTC1-6 after transfection with mimics of miR-296-3p or miR-298-5p, as compared to scramble-transfected controls; this decrease was higher than 3 folds when cells were transfected with both mimics (Figure 5A, left panel). The decrease of IGF1Rβ was not detectable in αTC1-6 transfected with either mimic of miR-296-3p or miR-298-5p and then treated with cytokines for 24 h. In αTC1-6 treated with cytokines for 24 h, after transfection with mimics of both miRNAs 296-3p and 298-5p, it was instead lower than at steady state (1.4 folds). This could also be due to the decrease of miRNAs 296-3p and 298-5p following treatment with cytokines (Figures 2B and 5A, middle panel). Following transfection of αTC1-6 with either mimics of miR-296-3p or miR-298-5p, TNFα protein was about 1.2 folds less expressed with respect to scramble-transfected controls; this decrease was more pronounced (1.6 folds) when cells were transfected with both mimics (Figure 5B, left panel). Each of the two miRNA mimics decreased the expression of TNFα more than 2 folds after 24 h of treatment with cytokines, but the silencing effect was not potentiated when the two mimics were transfected together (Figure 5B, middle panel).

### Activation of IRS-1 and ERK-1 is also under control of miR-296-3p and miR-298-5p

**Figure 6:**
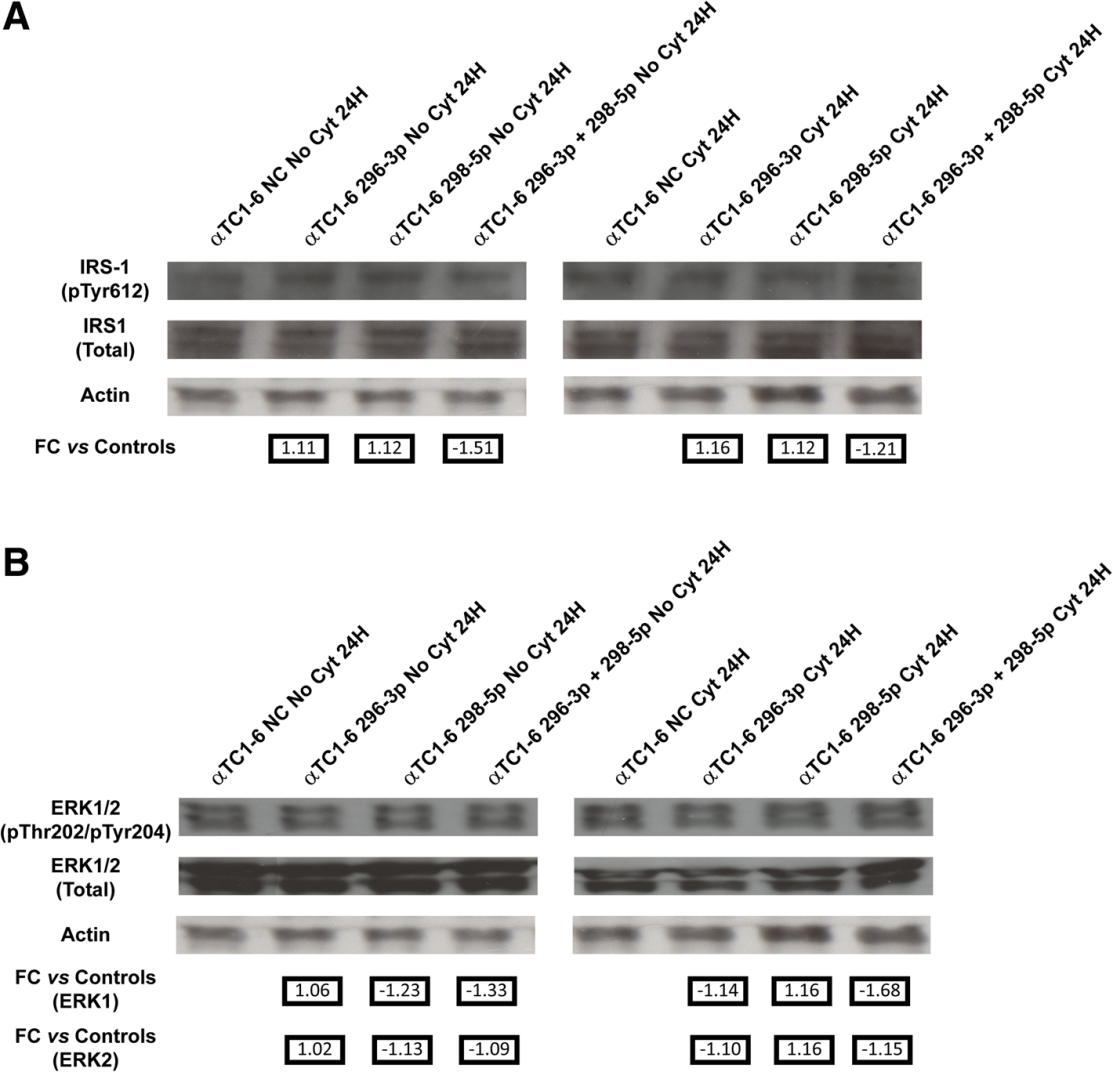
**Activation of IRS-1 and ERK-1 is under control of miR-296-3p and miR-298-5p in αTC1-6. (A)** Western analysis of phospho-IRS-1 (Tyr612) in (1) untreated αTC1-6 transfected for 24 h with (i) scramble molecules (NC); (ii) mimics of miR-296-3p; (iii) mimics of miR-298-5p; (iv) mimics of both miR-296-3p and miR-298-5p (left); (2) αTC1-6 transfected for 24 h with (i) scramble molecules (NC); (ii) mimics of miR-296-3p; (iii) mimics of miR-298-5p; (iv) mimics of both miR-296-3p and miR-298-5p and treated with cytokines for further 24 h (right). **(B)** Western analysis of phospho-ERK-1/2 (Thr202/Tyr204) performed in the same experimental conditions as in (**A**). Quantification of immunoblot signals was made by equalizing phospho-specific IRS-1 or Erk1/2 band intensities to total IRS-1 or Erk1/2, respectively. The decrease in phosphorylation was normalized to the basal level of the control and reported in arbitrary units as fold decrease over basal value. Numbers below Actin blots represent fold change expression values relative to matched controls.

To verify if the IGF1R signaling pathway was controlled by miR-296-3p and miR-298-5p through IGF1Rβ, we assayed the phosphorylation levels of IRS-1 and ERK (two markers downstream the IGF-1 receptor) in αTC1-6 transfected with mimics of each one or both miRNAs; this analysis was performed both after treatment with cytokines for 24 h or on untreated cells, exploiting as controls matched scramble-transfected αTC1-6 cells. The expression of phospho-IRS-1 didn’t change in untreated αTC1-6 transfected with mimics of each miRNA alone, while it decreased about 1.5 folds in αTC1-6 transfected with mimics of both miR-296-3p and miR-298-5p (Figure 6A, left panel). By using the same controls, we detected a slight decrease of phospho-IRS-1 (1.2 folds) in αTC1-6 transfected with mimics of both miR-296-3p and miR-298-5p and treated with cytokines (Figure 6A, right panel). Interestingly, also the activation of ERK-1 appears to be regulated by miR-296-3p and miR-298-5p: in the absence of treatment with cytokines, αTC1-6 cells transfected with mimics of miR-296-3p showed levels of phospho-ERK-1 (Thr202) similar to those found in scramble-transfected αTC1-6 cells; the transfection with mimics of miR-298-5p or of both miRNAs led instead to a decrease of the protein (1.2 and 1.3 folds, respectively) (Figure 6B, left panel). Under cytokine treatment, the amount of phospho-ERK-1 (Thr202) did not change in αTC1-6 transfected with either mimics of miR-296-3p or of miR-298-5p alone, compared to scramble-transfected cells; in αTC1-6 simultaneously transfected with mimics of both miRNAs, we detected a decrease of 1.7 folds of phospho-ERK-1 (Thr202) levels, compared to scramble-transfected cells (Figure 6B, right panel).

### Identification of TFs regulating DE miRNAs

In the genomic region comprising the genes encoding miR-296-3p, miR-298-5p and *Nespas*, MatInspector identified Transcription Factor Binding Sites (TFBS) for sixty seven Transcription Factors (TFs). Four of them (HMX2, HNF4A, LEF1, MAFB) are known to be expressed in the islets of Langerhans, and MAFB is known to be expressed only in rodent islet α cells within adult pancreas [25]; the presence of binding sites for this TF within the promoter of the genes encoding miR-296-3p and miR-298-5p suggests that it may regulate the expression of both miRNAs. Multi Experiment Matrix (MEM) showed a statistically highly significant negative correlation (p-values < 0.0001, Pearson Correlation test) between Mafb and Igf1r. Interestingly, Microrna.org predicts two binding sites for mmu-miR-296-3p and mmu-miR-298-5p on Mafb mRNA 3’ UTR.

### Differences between αTC1-6 and βTC1 steady state regulatory networks

14 of 73 miRNAs, more abundantly or exclusively expressed at steady state in αTC1-6 with respect to βTC1, have validated targets known to be expressed in mammalian pancreatic α cells (see link to T1Dbase in Materials and Methods). By using these data, we generated a network comprised of 560 nodes and 8222 edges (see Additional file 9): from this network, we inferred a subnetwork made of 4 α-miRNAs for which validated targets have been identified, consisting of 117 nodes and 530 edges (see Additional file 10). In the case of βTC1 cells, we found that 36 of 100 miRNAs more abundantly or exclusively expressed with respect to αTC1-6 have validated targets known to be expressed in pancreatic β cells (see link to T1Dbase): a network of 439 nodes and 2079 edges was generated from them (see Additional file 11). The subnetwork made of 15 β-miRNAs consisted of 107 nodes and 132 edges (see Additional file 12). The enrichment in specific biological processes was calculated in pancreatic αTC1-6 versus βTC1 cells subnetworks (networks made of genes regulated by miRNAs specifically expressed at steady state in αTC1-6 and βTC1 cells, respectively) (Table 1). Interestingly, in the network of genes regulated by α-miRNAs, *pancreatic α cell fate commitment* is a biological process overrepresented with respect to the network regulated by β-miRNAs (Hypergeometric test, Benjamini-Hochberg adjusted p-value < 0.05). Within the network of genes regulated by α-miRNAs, *insulin-like growth factor receptor signaling pathway* is a biological process significantly enriched among the genes interacting with targets of miR-296-3p and miR-298-5p, with respect to the genes linked to the targets of the other miRNAs (p-value = 0.039, Fisher’s exact test).

**Table 1:** Top 5 biological processes significantly enriched in α-miRNA networks with respect to β-miRNA networks

**Biological process**	**Adj-p-value (Benjamini-Hochberg)**
DNA replication	3.15E-23
Cell division	2.51E-09
Cellular response to metal ion	4.61E-05
Positive regulation of mesenchymal cell proliferation	5.24E-05
Pancreatic α cell fate commitment	2.23E-02

Biological processes are ranked based on increasing adjusted p-values (from the most to the least significant biological process).

## Discussion

The data reported in this paper and those from the literature [12] confirm that mammalian pancreatic α cells are more resistant than β cells to apoptosis induced by cytokines (Figures 1A and 1B). Our results suggest that miR-296-3p and miR-298-5p play a pivotal role in determining this trait. Due to DM *epidemic* spreading [26], its molecular characterization has become an important biological and translational problem in BioMedicine [7]. Our HT and network analyses of miRNA transcriptome in α and β cells highlighted very different profiles at steady state (see Table 1, Additional file 1). Computational analysis of these data allowed us to identify a set of miRNAs, specifically synthesized in pancreatic α cells, which appear to also regulate pancreatic α cell commitment. Specific enrichment of pathways, regulated by α-miRNAs within this biological process (Table 1), allowed the identification of miRNAs that could be involved in the acquisition of the trait *resistance to cytokines-induced cell death* upon pancreatic α cells differentiation. Among them, miR-296-3p and miR-298-5p stood out clearly as potentially critical nodes, responsible for α cells resistance to cytokine-induced cell death. In fact, besides being specifically expressed at steady state in α cells (αTC1-6), but not in β cells (βTC1 or INS-1), both miRNAs were significantly co-expressed and downregulated in αTC1-6 during a time-course treatment with cytokines, compared to matched untreated controls (see Figures 2A and 2B, Additional file 6). Our subsequent functional assays demonstrated that αTC1-6 transfected with mimics of miR-296-3p and miR-298-5p became susceptible to apoptosis when treated with the same cocktail of cytokines as compared to αTC1-6 transfected with scramble molecules. Although its biological effect was potentiated when also miR-296-3p was expressed, our results suggest that the role of miR-298-5p is more important in this process than that of miR-296-3p (Figure 3): this would highlight the role of one or a few specific miR-298-5p targets. Through western analysis, we confirmed our computational prediction that IGF1Rβ and TNFα are common targets to both miRNAs and that miR-296-3p and miR-298-5p also control IRS-1 and ERK-1 within the IGF1R signaling pathway. IGF1R is known to promote resistance to apoptosis by different mechanisms: it increases the levels of antiapoptotic proteins, as BCL2 and BCL-XL; it inactivates proapoptotic proteins, as BAD and CASP9; it stimulates mitogenic IRS-1/MAPKs pathway [27-29]. Phospho-IRS1 (Tyr612) and Phospho-ERK-1 (Thr202), which we demonstrated to be controlled by miR-296-3p and miR-298-5p, are known to regulate the response to insulin and to be involved in survival and proliferation processes [30]. Decreased expression of mir-296-3p and miR-298-5p and the corresponding activation of survival and proliferation signals, mediated by IGF1R and its downstream nodes (e.g., IRS-1 and ERK-1), may thus explain why αTC1-6 cells are resistant to death induction by cytokines (see Additional file 13). TNFα is known to be physiologically expressed by pancreatic endocrine cells and to contribute to maintain islet homeostasis [5,31]. This protein is also known to decrease apoptosis levels of pancreatic acinar cells during acute pancreatitis by stimulating the synthesis of antiapoptotic proteins [32].

## Conclusions

HT miRNA profiling data, functional analysis with synthetic mimics and molecular characterisation of modulated pathways strongly suggest that specific downregulation of miR-296-3p and miR-298-5p in pancreatic α cells, coupled to upregulation of their targets as IGF1Rβ and TNFα and activation of the corresponding signaling pathways, is a major determinant of their resistance to apoptosis induction by cytokines. Studies on pancreatic islet microorgan *in toto* will allow to experimentally confirm these results in a 3D natural system. It also will permit to verify the hypothesis that increased TNFα synthesis by α cells during insulitis protects them by activating survival pathways, while priming a deadly regulatory loop and causing β cells apoptosis (see Additional file 13).

## Methods

### Cell culture and treatment with cytokines

Mouse glucagonoma cell line αTC1-6 was obtained from the American Type Culture Collection (ATCC); it was cultured in complete Dulbecco’s modified Eagle’s medium (DMEM, Sigma-Aldrich^®^, Saint Louis, MO, USA), as described [33]. Mouse insulinoma cell line βTC1 also was from ATCC; cells were grown in DMEM with 25 mM glucose (Sigma-Aldrich^®^), supplemented with 2 mM L-Glutamine, 15% heat inactivated (HI) horse serum, 2.5% HI fetal bovine serum (FBS), 1% penicillin/streptomycin, in 95% humidified air-5% CO_2_ at 37° C. Cells were passaged once a week after trypsinization and replaced with new medium twice weekly. Treatment with cytokines (recombinant murine IL-1β, specific activity 5 × 10^8^ U/mg, Preprotech, London, UK, UE; recombinant murine IFN-γ, specific activity 1 × 10^7^ U/mg, Preprotech; recombinant murine TNF-α, specific activity 1 × 10^7^ U/mg, Preprotech) was as described [34]. αTC1-6 cells (passages 20–40) were seeded the day before treatment in 60 mm dishes at a density of 4.5 × 10^5^ cells.

### Evaluation of apoptosis and necrosis

Percentage of apoptotic or necrotic cells was assessed through flow cytometry. Analysis was performed with a Beckman Coulter Epics XL-MCL flow cytometer (Beckman Coulter^©^, Inc., Hialeah, FL, USA). Cells were collected, washed with phosphate-buffered saline (PBS), and stained with Annexin V-FITC/propidium iodide (PI) (Sigma-Aldrich^®^) in Annexin-V binding buffer, as specified by the manufacturer. EXPO32 ADC Analysis™ software package (Beckman Coulter^©^, Inc.) was used for data analysis.

### RNA extraction and HT quantitative RT-PCR

Total RNA was extracted with Trizol (Lifetechnologies™, Foster-City, CA, USA), according to manufacturer’s instructions. RNA quantification was performed with a Qubit^®^ Fluorometer (Lifetechnologies™). RNA for HT miRNA expression profiling was reverse transcribed into cDNA of 519 mouse-specific and 68 rat-specific miRNAs through Megaplex™ RT Rodent Primer Pool sets, and preamplified through Megaplex™ PreAmp Rodent Primer Pool sets (Lifetechnologies™). Resulting cDNAs were loaded onto TaqMan Low Density miRNA Arrays (TLDA) cards, according to manufacturer’s instructions. TLDA cards were run on ABI 7900HT Real Time PCR system. RNA for Single TaqMan miRNA Assays was reverse transcribed into miRNA-specific cDNA through TaqMan^®^ MicroRNA Reverse Transcription Kit (Lifetechnologies™) and amplified using TaqMan^®^ Universal Master Mix (Lifetechnologies™), according to manufacturer’s instruction. RNA for analysis of miRNA targets was reverse transcribed into cDNA through High Capacity RNA-to-cDNA Kit (Lifetechnologies™) and amplified through Fast SYBR^®^ Green Master Mix (Lifetechnologies™), according to manufacturer’s instruction. Primer sequences are available upon request.

### Criteria for selecting downregulated or overexpressed miRNAs

Data quality and quantification were computed using Real-Time Statminer^®^ software (http://www.integromics.com) (Integromics, Granada, Spain). Multiple reference genes were used to normalize data: Genorm (integrated into Real-Time Statminer^®^) [35] and DataAssist™ (Lifetechnologies™) softwares allowed to choose the best ones. Limma test (see below: *Statistical analysis*) was carried out by Real-Time Statminer^®^ to assess statistically significant DE genes. DE miRNAs were ranked based on their p-values and adjusted p-values (Benjamini-Hochberg correction with False Discovery Rate, FDR, set at 5%). Relative quantities (RQ) of miRNAs between αTC1-6 and βTC1, at steady state as between treated αTC1-6 cells and matched untreated controls, were calculated according to 2^-ΔΔCt^ method [36]. Values are reported as average fold changes of three independent biological replicates; RQ values < 1 were converted to negative fold changes by the formula: -1/RQ. The data files for each array are publicly available at the Gene Expression Omnibus (GEO) database repository (http://www.ncbi.nlm.nih.gov/geo/) (GSE42970).

### Transient transfection of αTC1-6 cells with mimics of miR-296-3p and miR-298-5p

For transfection, αTC1-6 cells were plated into 24-well plates at a density of 4×10^4^ cells per well to obtain RNA, and into 100 mm dishes at a density of 1.15×10^6^ cells to obtain proteins. Transfections were performed using siPORT™ NeoFX™ (Lifetechnologies™) with 30 nM mimics of miR-296-3p/miR-298-5p/scrambled sequence (Pre-miR™ miRNA Precursor Molecules—Negative Control #1, Lifetechnologies™). For each experiment, efficiency of transfection was measured through real-time PCR.

### *In silico* identification of miRNA targets

Validated targets of DE miRNAs were retrieved from the literature and miRTarbase (release 2.5) [37]. Predictions were performed through starBase (release 2.1) (http://starbase.sysu.edu.cn/). Among validated and predicted targets, only genes expressed in pancreatic cells (data from *Beta Cell Gene Atlas*, found at http://t1dbase.org/page/AtlasHome) and known to be functionally involved in cell survival or death were chosen for real-time PCR and western blot assays. Data on genomic position of genes encoding miRNAs and their assignment to specific families were from MiRBase (http://www.mirbase.org/).

### Western analysis

Protein lysates and their quantification were obtained as previously described [38]. 50 μg of total protein extract were loaded into 10% SDS polyacrylamide gel (Hoefer miniVE, GE Healthcare^©^, Amersham Place, Buckinghamshire, UK) and blotted to nitrocellulose membranes by iBlot Dry Blotting System (Lifetechnologies™). Membranes were probed with polyclonal antibodies to IGF1Rβ (Santa Cruz Biotechnology^®^, Inc., Dallas, TX, USA), p-IRS-1 (Santa Cruz Biotechnology^®^, Inc.), total-IRS-1 (EMD Millipore Corporation^©^, Billerica, MA, USA), p-p44/p42 MAPK (Cell Signaling Technology^®^, Inc., Danvers, MA, USA), total p44/p42 MAPK (Cell Signaling Technology^®^, Inc.), TNFα (Cell Signaling Technology^®^, Inc.), using β-actin (Sigma-Aldrich^®^) as loading control. Proteins were detected by using ECL™ Western Blotting Detection Reagents (GE Healthcare^©^). Densitometric analyses were performed by ImageJ software (http://rsbweb.nih.gov/ij/index.html)

### Prediction of transcription factors regulating expression of miRNAs 296-3p and 298-5p

MatInspector from Genomatix (http://www.genomatix.de/) was used to identify Transcription Factors Binding Sites (TFBS) and their corresponding Transcription Factors (TFs) [39]. By using Multi Experiment Matrix (MEM) (http://biit.cs.ut.ee/mem) (selected collection: Affymetrix GeneChip Mouse Genome 430 2.0 [Mouse_430_2] platform, used to analyze 1546 datasets), TFs prediction was interpolated with data on statistically significant expression correlation among TFs, which regulate DE miRNAs and their mRNA targets. Settings used in MEM are described in Additional file 14.

### Identification of CpG islands upstream the genes encoding Nespas, miR 296-3p and miR 298-5p

CpG islands upstream genes encoding miR-296, miR-298, *Nespas* were identified through UCSC Genome Browser (http://genome.ucsc.edu/).

### Network analysis

Biological networks comprising miRNAs, their predicted upstream regulators (TFs), their validated targets and first neighbours interactants, were generated by retrieving interactome data through MiMI Cytoscape plugin [40] and visualized by Cytoscape v. 2.8.1. Biological processes and pathways involving network nodes were analyzed through the tool DAVID (http://david.abcc.ncifcrf.gov/) and BiNGO Cytoscape plugin [41]

### Statistical analysis

P-values were calculated by applying different methods: Limma test [42], associated with Benjamini-Hochberg correction for multiple comparison, was applied to identify DE miRNA genes between test and control samples in HT miRNA transcriptome analyses; Student’s *t*-test, associated with Bonferroni correction method, was used to statistically analyze data from single TaqMan assays and to assess significantly different apoptotic levels between αTC1-6 treated with cytokines and their matched untreated controls; Tukey HSD post-hoc one-way ANOVA test was used to evaluate significant differences in apoptosis among different transfection experimental conditions. For analysis of correlation between the expression of miR-296-3p and miR-298-5p in αTC1-6, Pearson correlation coefficient was calculated. Finally, *χ*^2^-square test was used to establish if miR-296-3p and miR-298-5p have more common targets than expected by chance; Fisher’s exact test was applied to evaluate the enrichment in specific gene ontologies. All statistical tests and correction methods, used to calculate p-values, are described throughout the text and figure legends.

### Nomenclature of genes and proteins

Rules for official gene and protein symbols by the International Committee on Standardized Genetic Nomenclature for Mice were followed throughout [43] (http://www.informatics.jax.org/mgihome/nomen/gene.shtml).

## Abbreviations

AT: After transfection; DE: Differentially expressed; DM: Diabetes mellitus; GLP1: Glucagon-like peptide 1; HT: High-throughput; IFN-γ: Intereferon gamma; IL-1β: Interleukin 1 beta; miRNA: microRNA; Ngn3: Neurogenin 3; PT: Post treatment; STAs: Single TaqMan gene expression assays; T1DM: Type 1 diabetes mellitus; T2DM: Type 2 diabetes mellitus; TLDA: TaqMan Low Density Array; TNF-α: Tumor Necrosis Factor alpha; TSS: Transcription Start Site.

## Competing interests

The authors declare that they have no competing interests.

## Authors’ contributions

MP and FP conceived and coordinated the project; MP, FP, DB, SP, AMR, CDP designed experiments, DB, AGC, LGM, FU, NP, AM, LS performed them; MP, FP, DB wrote the paper; all authors contributed to the critical revision of the data, read and approved the final manuscript.

## Supplementary Material

Additional file 1 DE miRNAs between αTC1-6 and βTC1 at steady state. Click here for file

Additional file 2 Classification in families of miRNAs more abundantly or exclusively expressed in αTC1-6 respect to βTC1 at steady state. Click here for file

Additional file 3 Classification in families of miRNAs more abundantly or exclusively expressed in βTC1 with respect to αTC1-6 at steady state. Click here for file

Additional file 4 **αTC1-6 DE miRNAs after 24 and 48 h of treatment with cytokines.** In bold are indicated significant DE miRNAs (adjusted p-values < 0.05. Limma Test, Benjamini-Hochberg correction). MiRNAs are ranked based on increasing adjusted p-values (from the most to the least significant DE miRNAs). Click here for file

Additional file 5 Classification in families of DE miRNAs in αTC1-6 after treatment with cytokines. Click here for file

Additional file 6 **Scatter plot showing correlation between miR-296-3p (x-axis) and miR-298-5p (y-axis) expression in αTC1-6, during a 6-12-24-48 h time-course experiment.** For each time point DCt values of miR-296-3p and miR-298-5p were correlated, both from untreated and cytokines-treated αTC1-6 cells (r-value = 0.88, p-value = 1.15e-08, Pearson’s correlation test). Three independent biological replicates (n = 3) have been analyzed at each time point. Click here for file

Additional file 7 **On scale representation of the genome segment comprising*Nespas*, miR-296, miR-298.** CpG islands are indicated as red vertical lines; pre-miRNAs 296 and 298 are depicted as blue and green boxes, respectively; exons of noncoding RNA *Nespas* are shown as yellow boxes. Expression of a macro-noncoding RNA (precursor of miR-296, miR-298, *Nespas*) is predicted to be controlled by two groups of CpG islands (one comprising two CpG islands, from 17.5 to 18.8 kb upstream the first nucleotide of pre-miR-296; the other made of three CpG islands, from 30.1 to 33.6 Kb upstream the first nucleotide of pre-miR-296). Click here for file

Additional file 8 **Validated and predicted targets of miR-296-3p and miR-298-5p.** A selection of validated and predicted targets of miR-296-3p and miR-298-5p was chosen according to their involvement in apoptosis, cell cycle progression, cell differentiation and hormone secretion. Click here for file

Additional file 9 **Interaction network among miRNAs more abundantly expressed at steady state in αTC1-6 with respect to βTC1, their validated targets and first neighbours interactants.** The network generated from validated targets and first neighbours interactants of 14 out of 50 miRNAs, more abundantly expressed at steady state in αTC1-6 with respect to βTC1, consisted of 560 nodes and 8222 edges. MiRNAs are represented as fuchsia diamonds. This file can be opened and browsed through Cytoscape tool and its plugins (http://www.cytoscape.org/). Click here for file

Additional file 10 **Interaction network among miRNAs specifically expressed at steady state in αTC1-6 (α-miRNAs) with respect to βTC1, their validated targets and first neighbours interactants.** The network generated from validated targets and first neighbours interactants of 4 out of 23 α-miRNAs consisted of 117 nodes and 530 edges. MiRNAs are represented as fuchsia diamonds. This file can be opened and browsed through Cytoscape tool and its plugins (http://www.cytoscape.org/). Click here for file

Additional file 11 **Interaction network among miRNAs more abundantly expressed at steady state in βTC1 with respect to αTC1-6, their validated targets and first neighbours interactants.** The network generated from validated targets and first neighbours interactants of 36 of 74 miRNAs more abundantly expressed at steady state in βTC1 with respect to αTC1-6 consisted of 439 nodes and 2079 edges. MiRNAs are represented as fuchsia diamonds. This file can be opened and browsed through Cytoscape tool and its plugins (http://www.cytoscape.org/). Click here for file

Additional file 12 **Interaction network among miRNAs specifically expressed at steady state in βTC1-6 (β-miRNAs) with respect to αTC1-6, their validated targets and first neighbours interactants.** The network generated from validated targets and first neighbours interactants of 15 of 26 β-miRNAs consisted of 107 nodes and 132 edges. MiRNAs are represented as fuchsia diamonds. This file can be opened and browsed through Cytoscape tool and its plugins (http://www.cytoscape.org/). Click here for file

Additional file 13 Hypothetical model of regulation of miR-296-3p and miR-298-5p biomolecular activity in αTC1-6 at steady state (left) and after treatment with cytokines (right). Click here for file

Additional file 14 Additional Methods. Click here for file
