# Supplementary figures and images for: miR-296-3p, miR-298-5p and their downstream networks are causally involved in the higher resistance of mammalian pancreatic α cells to cytokine-induced apoptosis as compared to β cells
Source: BMC Genomics. 2013 Jan 29;14:62. doi: 10.1186/1471-2164-14-62 (PMC3571888; doi:10.1186/1471-2164-14-62)

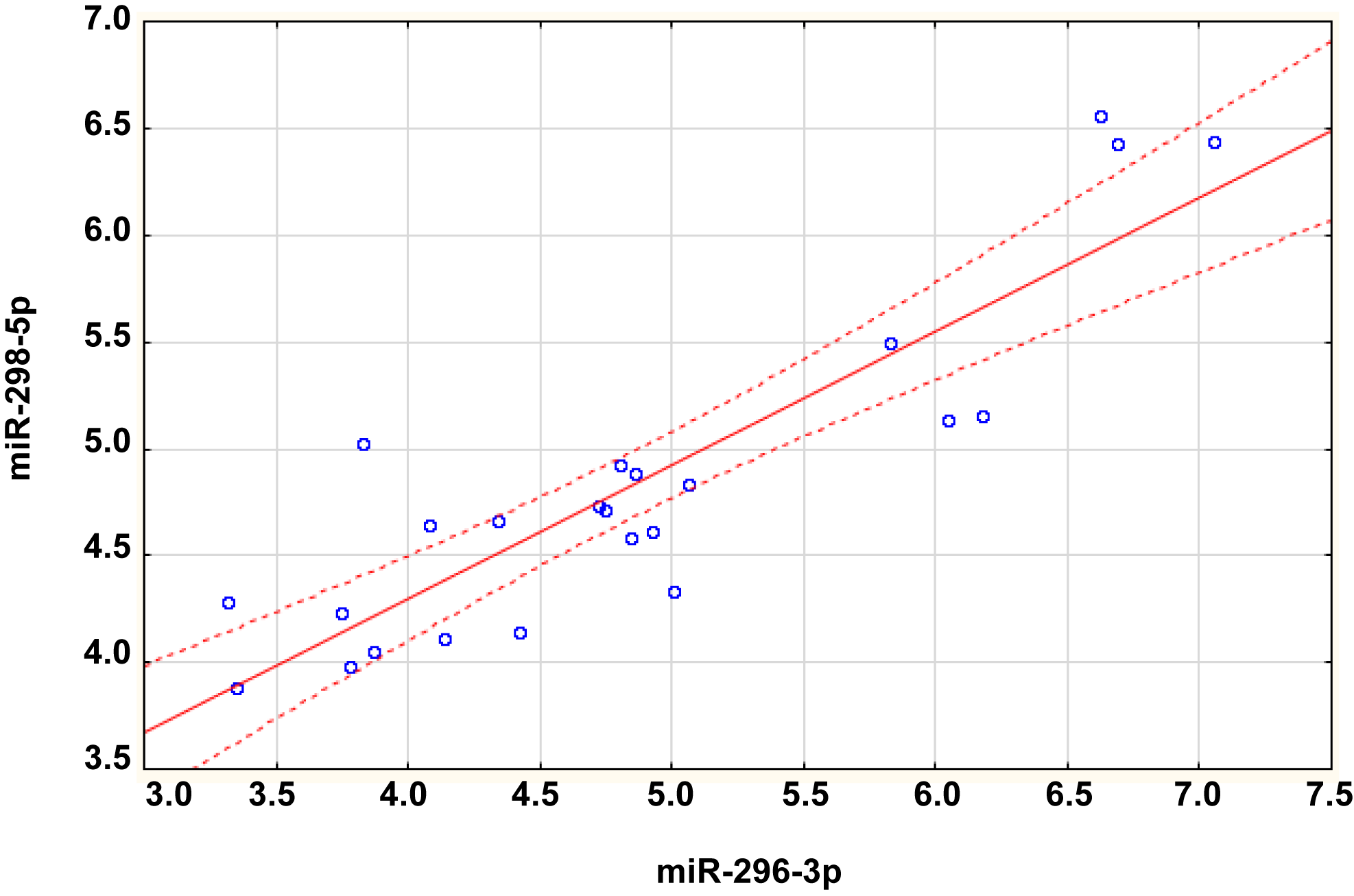

Supplement: Additional file 6 — Scatter plot showing correlation between miR-296-3p (x-axis) and miR-298-5p (y-axis) expression in αTC1-6, during a 6-12-24-48 h time-course experiment. For each time point DCt values of miR-296-3p and miR-298-5p were correlated, both from untreated and cytokines-treated αTC1-6 cells (r-value = 0.88, p-value = 1.15e-08, Pearson’s correlation test). Three independent biological replicates (n = 3) have been analyzed at each time point. [file 1471-2164-14-62-S6.tiff]

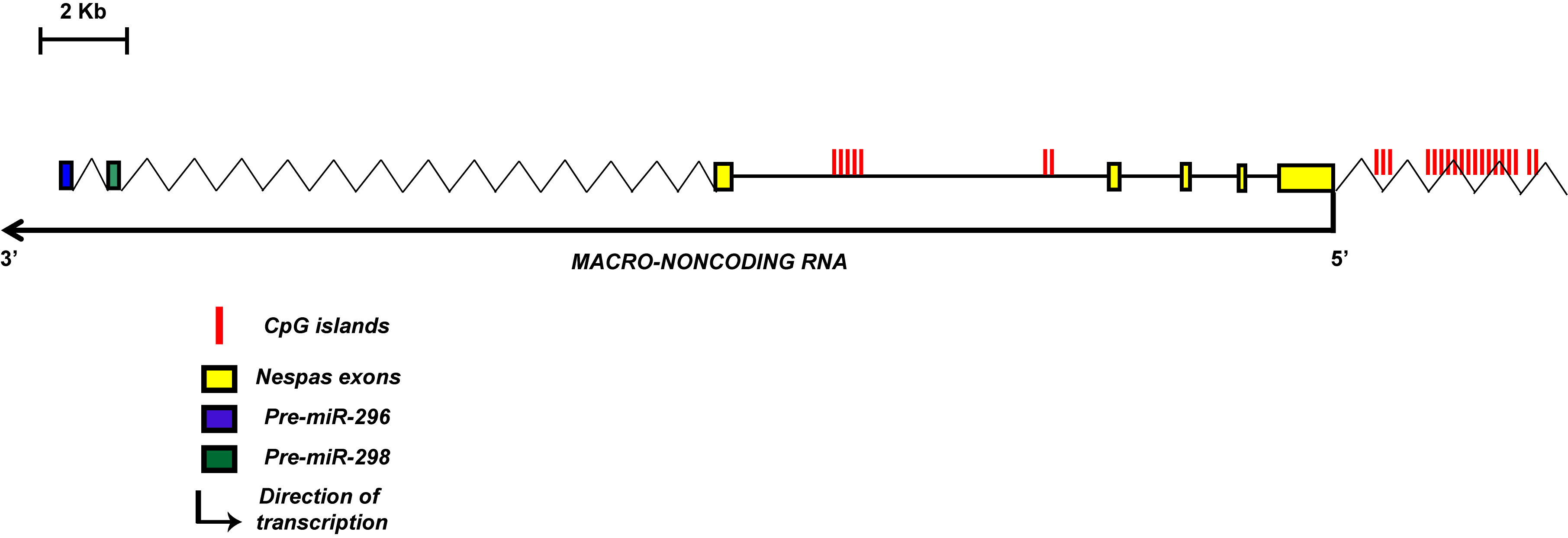

Supplement: Additional file 7 — On scale representation of the genome segment comprisingNespas, miR-296, miR-298. CpG islands are indicated as red vertical lines; pre-miRNAs 296 and 298 are depicted as blue and green boxes, respectively; exons of noncoding RNA Nespas are shown as yellow boxes. Expression of a macro-noncoding RNA (precursor of miR-296, miR-298, Nespas) is predicted to be controlled by two groups of CpG islands (one comprising two CpG islands, from 17.5 to 18.8 kb upstream the first nucleotide of pre-miR-296; the other made of three CpG islands, from 30.1 to 33.6 Kb upstream the first nucleotide of pre-miR-296). [file 1471-2164-14-62-S7.tiff]

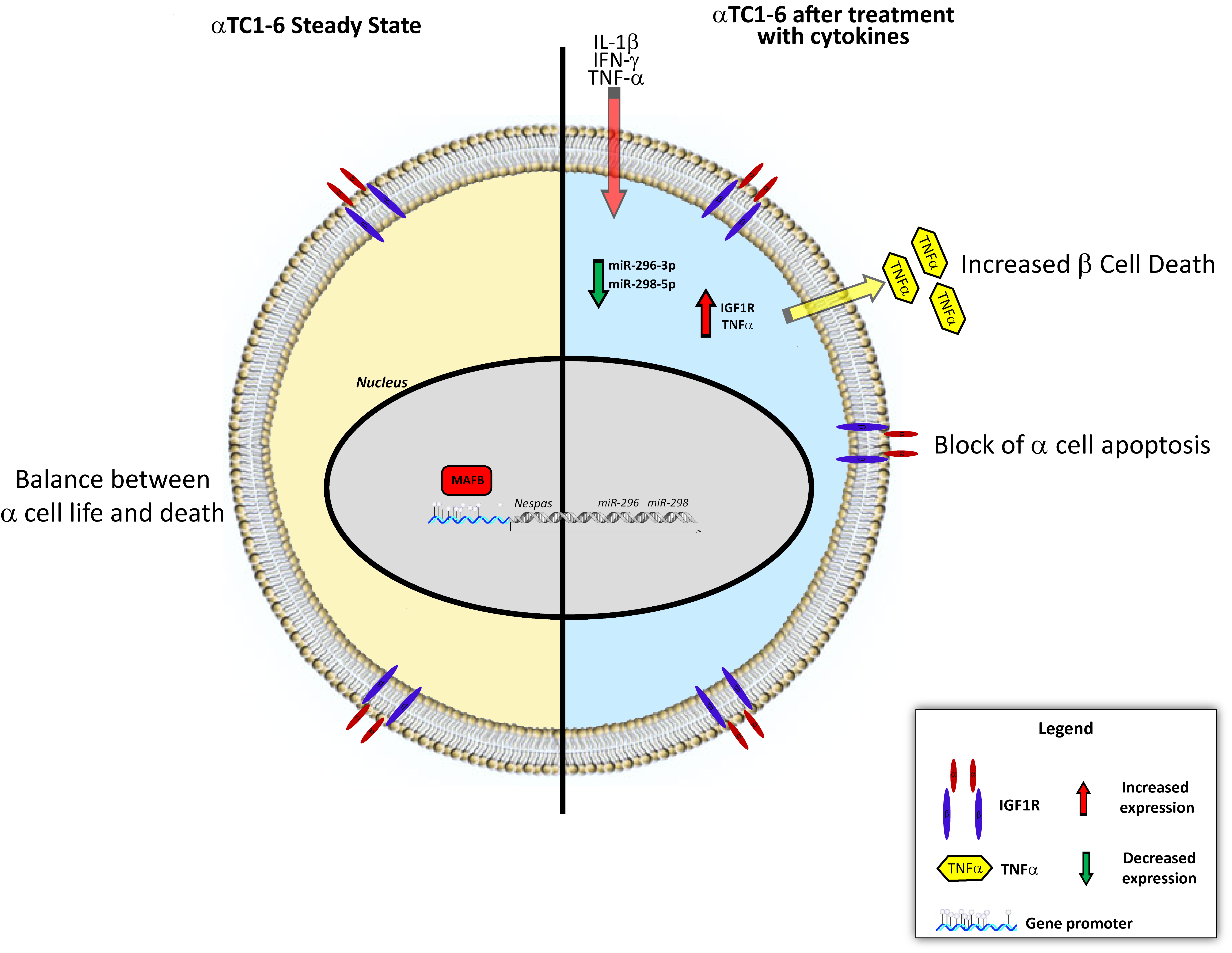

Supplement: Additional file 13 — Hypothetical model of regulation of miR-296-3p and miR-298-5p biomolecular activity in αTC1-6 at steady state (left) and after treatment with cytokines (right). [file 1471-2164-14-62-S13.tiff]
