# Supplementary material for: miR-296-3p, miR-298-5p and their downstream networks are causally involved in the higher resistance of mammalian pancreatic α cells to cytokine-induced apoptosis as compared to β cells
Source: BMC Genomics. 2013 Jan 29;14:62. doi: 10.1186/1471-2164-14-62 (PMC3571888; doi:10.1186/1471-2164-14-62)
Supplement: Additional file 14 — Additional Methods. [file 1471-2164-14-62-S14.pdf]

**Additional Methods - MEM (Multi Experiment Matrix) Settings**

Following parameters were used in MEM. As collection, we selected Affymetrix GeneChip Mouse Genome 430 2.0 [Mouse_430_2] platform (1546 datasets). From “Options” parameters in “Similarity” section we selected Pearson correlation distance as Distance Method; Beta MEM method to get p-value for selected ranks as Rank aggregation method; threshold score was set as “inf” and most distant genes were displayed. In “Data filters” section we set standard deviation threshold for query gene to 0.29; the most variant datasets with respect to query genes were set at 100. We considered as positively or negatively correlated only genes that had all the probes representing them positively or negatively correlated, respectively. We discarded all ambiguous correlations (e.g., couples of genes for which only a subset of probes significantly correlated or couple of genes whose probes both positively and negatively correlated at the same time).
